# The Physico-Chemical Properties and Exploratory Real-Time Cell Analysis of Hydroxyapatite Nanopowders Substituted with Ce, Mg, Sr, and Zn (0.5–5 at.%)

**DOI:** 10.3390/ma14143808

**Published:** 2021-07-08

**Authors:** Iuliana Maria Chirică, Ana-Maria Enciu, Teddy Tite, Maria Dudău, Lucian Albulescu, Simona Liliana Iconaru, Daniela Predoi, Iuliana Pasuk, Monica Enculescu, Cristian Radu, Cătălina Gabriela Mihalcea, Adrian-Claudiu Popa, Nicoleta Rusu, Sultana Niţă, Cristiana Tănase, George E. Stan

**Affiliations:** 1 National Institute of Materials Physics, RO-077125 Măgurele, Romania; iuliana.bogdan@infim.ro (I.M.C.); simonaiconaru@gmail.com (S.L.I.); dpredoi@gmail.com (D.P.); iuliana.pasuk@infim.ro (I.P.); mdatcu@infim.ro (M.E.); cristian.radu@infim.ro (C.R.); catalina.mihalcea@infim.ro (C.G.M.); adrian.popa@gmail.com (A.-C.P.); 2 Faculty of Physics, University of Bucharest, RO-077125 Măgurele, Romania; 3 “Victor Babes” National Institute of Pathology, RO-050096 Bucharest, Romania; ana.enciu@ivb.ro (A.-M.E.); maria.dudau@drd.umfcd.ro (M.D.); lucian.albulescu@ivb.ro (L.A.); 4 Department of Cellular Biology and Histology, “Carol Davila” University of Medicine and Pharmacy, RO-050047 Bucharest, Romania; 5 National Institute for Chemical Pharmaceutical Research and Development, RO-031299 Bucharest, Romania; ninarusu55@yahoo.com (N.R.); sultananita@yahoo.com (S.N.); 6 “Nicolae Cajal” Institute, “Titu Maiorescu” University, RO-004051 Bucharest, Romania

**Keywords:** substituted hydroxyapatite, structure, composition, bio-functionality, ICP-MS, biocompatibility, real-time cell analysis

## Abstract

Cation-substituted hydroxyapatite (HA), standalone or as a composite (blended with polymers or metals), is currently regarded as a noteworthy candidate material for bone repair/regeneration either in the form of powders, porous scaffolds or coatings for endo-osseous dental and orthopaedic implants. As a response to the numerous contradictions reported in literature, this work presents, in one study, the physico-chemical properties and the cytocompatibility response of single cation-doped (Ce, Mg, Sr or Zn) HA nanopowders in a wide concentration range (0.5–5 at.%). The modification of composition, morphology, and structure was multiparametrically monitored via energy dispersive X-ray, X-ray photoelectron, Fourier-transform infrared and micro-Raman spectroscopy methods, as well as by transmission electron microscopy and X-ray diffraction. From a compositional point of view, Ce and Sr were well-incorporated in HA, while slight and pronounced deviations were observed for Mg and Zn, respectively. The change of the lattice parameters, crystallite size, and substituting cation occupation factors either in the Ca(I) or Ca(II) sites were further determined. Sr produced the most important HA structural changes. The in vitro biological performance was evaluated by the (i) determination of leached therapeutic cations (by inductively coupled plasma mass spectrometry) and (ii) assessment of cell behaviour by both conventional assays (e.g., proliferation—3-(4,5-dimethyl thiazol-2-yl) 5-(3-carboxymethoxyphenyl)-2-(4-sulfophenyl)-2H-tetrazolium assay; cytotoxicity—lactate dehydrogenase release assay) and, for the first time, real-time cell analysis (RTCA). Three cell lines were employed: fibroblast, osteoblast, and endothelial. When monophasic, the substituted HA supported the cells’ viability and proliferation without signs of toxicity. The RTCA results indicate the excellent adherence of cells. The study strived to offer a perspective on the behaviour of Ce-, Mg-, Sr-, or Zn-substituted HAs and to deliver a well-encompassing viewpoint on their effects. This can be highly important for the future development of such bioceramics, paving the road toward the identification of candidates with highly promising therapeutic effects.

## 1. Introduction

The development of high-performance biomaterials capable not only of treating, but also reinforcing or even replacing body parts or living tissues, is nowadays of paramount importance for the healthcare and tissue-engineering fields [1]. Biomaterials used in healthcare can be classified into metals, polymers, ceramics, and composites [2,3]. One prominent category of current clinical applications focuses on bioceramics for hard conjunctive tissue (e.g., bone and teeth) regeneration [1,2,3], with emphasis on the design of bone grafting scaffolds and biofunctionalisation, as well as improvement and prolongation of the lifetime of endo-osseous/dental implants. Hydroxyapatite (HA)-based materials are currently the main exponent of the bioceramics family. The pioneering use in clinical practice of synthetic HA can be dated back to the 1950s [4] and was inspired by the mineral composition of bone [1]. Its ongoing success is related to its unique physico-chemical properties and its excellent biofunctionality (e.g., biocompatibility, bioactivity, and osteoconductivity) [1,5,6,7]. HA particularities have been highlighted in various biomedical applications, especially in dentistry and orthopaedics, as implant coatings [8], bone graft substitutes [9], drug delivery systems [10,11], components of composite implants [12], or dental fillers [13]. The general interest for the HA field is demonstrated also by the progressive yearly increase of topical scientific articles [5,14].

Synthetic HA exists either in stoichiometric or nonstoichiometric form. However, synthetic stoichiometric HA (Ca_10_(PO_4_)_6_(OH)_2_ with a theoretical Ca/P molar ratio of 1.67) has poor mechanical properties [1,15,16] and thus is not indicated as a standalone material for load-bearing applications. One of the primary fascinating aspects of HA is its ability to incorporate fairly large amounts of foreign elements (as both cations and/or anions) within its structure, thus fostering the creation of designed nonstoichiometric forms. Actually, biological apatites are themselves carbonated Ca-deficient apatites with trace amounts of various cations and anions [1]. Therefore, the production of substituted hydroxyapatite (SHA), by either synthetic or non-synthetic routes [5], has recently attracted a lot of interest since SHAs are able to mimic the composition of the bone and teeth mineral component. Furthermore, SHAs could present superior mechanical properties and biological performance with respect to pure stoichiometric HA [5,6]. For instance, the fracture toughness (K_1c_) of pure HA, situated in the range of 0.5–1 MPa.m^1/2^, was improved up to 2.7 MPa·m^1/2^ by partial Ca substitution with 0.6 wt.% of Mg [17].

Cationic substitution involves the fractional replacement of the bivalent calcium (Ca) ions in the HA crystalline lattice by the monovalent (e.g., lithium (Li) [18,19], sodium (Na) [20,21], silver (Ag) [22,23]), bivalent (e.g., barium (Ba) [24,25], magnesium (Mg) [6,26,27], strontium (Sr) [28,29,30], copper (Cu) [31,32], zinc (Zn) [33,34,35]), or multivalent (e.g., aluminium (Al) [36], cerium (Ce) [37,38,39], gallium (Ga) [40], or titanium (Ti) [41,42]) ion species, either in the Ca(I) or Ca(II) sites [5]. In the prospect of this study, it is worth noting that cation-substituted HA, which is easier to synthesise than the anion-substituted one, yields the most prominent therapeutic effects [1,6]. Although synthetic methods are costlier than non-synthetic routes (i.e., employing natural and biological resources), they offer the possibility to fine-tune the properties of cation-substituted HA in order to enhance HA specificities (i.e., toward patient) and long-term performance.

On a general level, the cationic substitution in HA has been intensively explored for various scientific and technical purposes, with the type and amount of dopants being situated in a notably wide range [5]. However, only some elements have been suggested to possess biofunctional effects. For instance, Ce, Sr, Zn, and Mg enhance the osteogenic capacity; Ce and Co have angiogenic activity; whilst cations such as Ag, Zn, and Ce possess antimicrobial efficacy [5,6,7]. Mg, Sr, and Zn are amongst the most studied substitution cations in HA, being also naturally found in bones and teeth as trace elements with essential roles on the health of these particular tissues [5,6,7]. Mg has been found to prevent osteoporosis and foster the calcification process and recovery/regeneration of damaged tissues [6,43]; its deficiency causes a decrease in the osteoblast and osteoclast activities [6,7,44]. Sr seems to play a major role in the biomineralisation of hard tissues, improving bone strength, and also being known as adjuvant in the treatment of osteoporosis (in the form of strontium ranelate), preventing bone loss by an inhibited osteoclast activity [1,5,6]. Zn is an important ion for controlling cell growth and differentiation [1,5,6]. Although, less studied, Ce seems to possess antioxidant properties toward noxious intracellular reactive oxygen species [45,46]. The in vivo biological performance of SHA-based materials relies on their positive interaction after implantation with the surrounding tissues. In vivo tests carried out on rabbits or rats using granulated SHA as filler for defective sites (e.g., femoral bone fracture) showed enhanced osteoconductivity and resorption compared to stoichiometric HA after being doped with Mg, Sr, or Zn [5]. Nowadays, it is of great importance to better understand the properties of HA substituted with Ce, Mg, Sr, and Zn since their combination could be an excellent strategy to synergistically expand SHA biofunctionality and better mimic the properties of biological apatites. This can unlock significant developments in the orthopaedic and dentistry fields.

However, despite the rich scientific literature and efforts made, disagreements over the physico-chemical properties of SHAs are evident, being linked to a rather systemic inhomogeneity in what concerns their synthesis and testing protocols [1,6,47,48]. Furthermore, it is important to emphasise that the biomedical application of SHAs is a rather new research field that currently generates quite evident discrepancies in what concerns the biofunctional effects and substituent dosage levels at which they can be induced [5,49]. Last but not least, the SHA field seems to be marred by a propensity for a “scientific salami slicing” philosophy and not one oriented toward “healthier” simultaneous comparative studies of the effect of more than one type of ion at various concentrations. For example, differences in the concentration of substituents and responses of bioceramics have been reported, depending on the synthesis technique and/or the biological assays used [5,6].

This is why the current joint efforts of the biomaterials research community aiming to delineate innovative biomaterials for bone regeneration (here including biofunctional advanced coatings [50,51] and scaffolds [52,53,54,55,56] based on SHAs) should be always accompanied by systematic and comprehensive comparative studies. Abiding to this strategy, the aim of this work was to compare in a single study, for the first time, the physico-chemical properties and the preliminary biological responses of HA substituted with the most promising cations. Pure and single-cation substituted (Ce, Mg, Sr, or Zn) nanopowders have been synthesised using the coprecipitation method, and their morphological, compositional, and structural properties were multiparametrically investigated, while their biological responses were assessed in vitro by various tests covering the (i) therapeutic ion-release determinations and (ii) study of cell behaviour by classical viability and cytotoxicity assays, as well as the not yet explored real-time cell analysis (RTCA) method. This work could pave the way for future developments in this field of knowledge, supporting a more decisive delineation of HA-based materials with controlled (both as intensity and duration) therapeutic effects (e.g., osteogenesis, angiogenesis, extended antimicrobial activity) suitable for the realisation of a safer and more effective generation of bone graft substitutes and implants.

## 2. Materials and Methods

### 2.1. Preparation of the HA-Based Materials: From Synthesis to Optimisation

The coprecipitation method was used to synthesise pure and substituted HA samples with Ce, Mg, Sr, and Zn due to its recognised advantages (e.g., control over the particle size, simplicity, lower fabrication costs and industrial scalability) [5,6].

An optimised preparation protocol has been achieved by investigating the influence of the main synthesis parameters (e.g., reagent concentration, pH of the reaction medium, precipitation time, reaction temperature, post-synthesis calcination) on the composition and structure of pure and substituted HA. For obtaining a batch of 12 g of pure HA, 9.433 g of (NH_4_)_2_HPO_4_ was introduced dropwise into the solution containing 28.133 g of Ca(NO_3_)_2_·4H_2_O under continuous stirring and with constant pH monitoring, kept in the range of 10−10.5. For SHA powders, cation (Me) doped with Ce, Mg, Sr, or Zn (at substitution levels of 0.5–5 at.% with respect to Ca), the following reagents Mg(NO_3_)_2_·6H_2_O, Zn(NO_3_)_2_·6H_2_O, Sr(NO_3_)_2_, and Ce(NO_3_)_3_·6H_2_O (purity: 99.999%, Alpha Aesar, Haverhill, MA, USA) were additionally used, in quantities reported to Ca, by keeping the (Ca+Me)/P molar ratio to 1.667 (the stoichiometric value of HA).

The resulting mixture was left under continuous stirring for 24 h and the precipitate was vacuum filtered, washed with bidistilled water, and dried under vacuum at 80 °C for ~24 h. For the synthesis of SHA, an additional step was required: the nitrate of Sr/Mg/Zn/Ce dissolved in bidistilled water was added to the solution containing calcium ions and then precipitated with ammonium phosphate according to the procedure described above. Nanopowders (NPs) of Ca_10-x_Me_x_(PO_4_)_6_(OH)_2_ with different concentrations of doping cations (x_M_ = 0.5, 1.0, 1.5, 2.5, and 5.0 at.%) were obtained. Subsequently, the product was milled and then heat-treated at 500 °C/2 h, as an integral part of the method’s adaptation process, such as to remove all residual (e.g., nitrite groups) reaction compounds (data not shown).

All HA and SHA NPs were physico-chemically and (in vitro) biologically characterised in freshly calcined form (never older than 1–2 weeks) to mitigate possible change in ambient. A standalone study on the time stability of HA-based nanomaterials is foreseen in the near future.

### 2.2. Physico-Chemical Characterisation Techniques

All physico-chemical analyses were performed at room temperature in ambient (Fourier-transform infrared (FTIR) and micro-Raman spectroscopy; X-ray diffraction (XRD)) or under vacuum (transmission electron microscopy (TEM); energy dispersive X-ray spectroscopy (EDXS); X-ray photoelectron spectroscopy (XPS)) conditions.

#### 2.2.1. Morphological Examination

The morpho-structural studies of the HA and SHA NPs were investigated via TEM (JEOL, model JEM-2100, Tokyo, Japan) using the conventional bright field mode. The TEM system, equipped with a LaB6 gun, was operated at an accelerating voltage of 80 kV in order to avoid beam damage. The specimens were prepared using a standard powder method: ~2 mg of NP was dispersed in 2 mL of ethanol and then ultrasonicated (Elmasonic S30H bath, Elma Schmidbauer GmbH, Singen, Germany) for ~10 min. The as-resulting solution was drop-casted on the TEM dedicated copper grid with carbon membrane (300 mesh, Agar Scientific Ltd., Stansted, UK). The grid was left for 30 min for the solvent to evaporate and then analysed.

#### 2.2.2. Compositional Analysis

For the quantitative composition analysis of SHA NPs, the EDXS method was elected since it allowed for a relevant comparison with respect to the scientific HA literature as EDXS stands to date as the most used analytical technique for this specific purpose. Furthermore, EDXS can be applied with confidence for the determination of mass fraction down to 1% for elements with the atomic number Z > 10, according to ISO 22309:2011: “Microbeam analysis—quantitative analysis using energy-dispersive spectrometry (EDS) for elements with an atomic number of 11 (Na) or above”. The EDXS measurements were performed in quadruplicate, averaging over different, randomly selected regions (having areas of ~700 × 480 µm^2^) of the specimens with the help of a Bruker 133 eV XFlash 4010 EDXS instrument (Bruker AXS Advanced X-ray Solutions GmbH, Karlsruhe, Germany) attached to a scanning electron microscope (Zeiss Evo 50 XVP, Jena, Germany). An acceleration voltage of 20 kV and a working distance of ~12 mm were used. The collected data were calibrated against National Institute of Standards and Technology (NIST) 2910b HA standard reference material (SRM).

#### 2.2.3. Chemical State Analysis

XPS was also carried out to unveil the chemical state of Ce in SHA. The spectra of the Ce 3d region has been acquired in the binding energy range 870–930 eV. The XPS measurements were performed with a Kratos analytical axis Ultra DLD (Kratos Analytical Ltd., Manchester, UK) with a monochromatic Mg K_α_ (1253.6 eV) radiation. All spectra were charge-corrected with respect to the adventitious carbon position (binding energy of 284.8 eV).

#### 2.2.4. Structural Investigation

FTIR spectroscopy represents a powerful analytical characterisation technique capable of disclosing the fine structural and chemical features of specimens. The FTIR spectroscopy measurements were performed with a PerkinElmer Spectrum BX II apparatus (PerkinElmer Corporation, Waltham, MA, USA) in attenuated total reflectance (ATR) mode using a Pike-MIRacle (PIKE Technologies, Madison, WI, USA) attachment with a diamond–zinc selenide crystal with a diameter of 1.8 mm. The spectra were registered in the wave numbers range 500–4000 cm^−1^ with a resolution of 4 cm^−1^ and a total of 32 scans per acquisition.

Complementary to FTIR-ATR, micro-Raman spectroscopy analyses were done in backscattering configuration with a LabRAM HR Evolution (Horiba Jobin-Yvon, Tokyo, Japan) device. A He–Ne laser radiation with a wavelength of 633 nm was employed and focused on the surface of the powder specimens with the help of an Olympus 100× objective. The scattered Raman signal was recorded with an 1800 lines/mm diffraction grating monochromator in the 350–1150 cm^−1^ spectral range at a resolution of ~0.5 cm^−1^ by using an acquisition time of 20 s and a total of 4 accumulations. The calibration of the micro-Raman spectra was carried out by using the Rayleigh (0 cm^−1^) and silicon (520.7 cm^−1^) standard bands.

The crystalline quality of the HA and SHA NPs was investigated via XRD with a Bruker D8 Advance diffractometer (Bruker AXS Advanced X-ray Solutions GmbH, Karlsruhe, Germany) with nickel-filtered CuK_α_ radiation (λ_Kα1_ = 1.5406 Å, 40 kV, 40 mA) and a high-efficiency one-dimensional detector with an angular window of 2.4° (LynxEye™ type) operating in integration mode. The goniometer optics consisted of: fixed divergence slit of 15 × 0.6 mm^2^ and axial Soller slit of 2.5° (for the incident beam) and antiscatter slit of 15 × 8 mm^2^ and axial Soller slit of 2.5° (for the diffracted beam). The spot size with this diffractometer setup was ~15 × 5 mm^2^. The HA and SHA NPs were loaded into sample holders with a cavity diameter of 25 mm and height of 2 mm. The patterns were recorded in Bragg–Brentano geometry in the 2θ range 9−70° with a step size of 0.02° and dwell time of 1 s. Quantitative analyses (i.e., determination of lattice parameters and cationic site occupation factors) were performed via Rietveld refinement using the MAUD diffraction data processing program (v2.55) by refining the anisotropic crystallite shapes of HA and SHAs. For comparison with the scientific literature, the average crystallite size (crystalline coherence length) along the *c*-axis was inferred by applying the prevalently/customarily used Scherrer equation [57]. The Scherrer method was implemented after removing the CuK_α2_ spectral line contribution using the Bruker-EVA software (Bruker AXS Advanced X-ray Solutions GmbH, Karlsruhe, Germany) based on the Rachinger algorithm. The width of the diffraction lines was further corrected for instrumental broadening using a corundum standard reference (i.e., NIST SRM 1976).

### 2.3. In Vitro Biological Assays

Prior to biological testing, all HA NPs were sterilized by gamma irradiation at the Multipurpose Irradiation Facility Centre, within the “Horia Hulubei” National Institute for R&D in Physics and Nuclear Engineering (IFIN-HH), Romania. The minimum absorbed dose was 25 kGy.

#### 2.3.1. Cation Release Determinations

Ion release tests were performed in the same medium as the cell culture assays, namely the Dulbecco’s Modified Eagle’s Medium/Nutrient Mixture F-12 Ham (DMEM/F12, D6421, Sigma-Aldrich, St. Louis, MO, USA) supplemented with 10% foetal bovine serum (FBS). This testing medium was selected instead of conventional inorganic solutions such as Kokubo’s simulated body fluid (SBF) or Tris-HCl (as suggested for instance in ISO 10993-14:2001: “Biological evaluation of medical devices—Part 14: Identification and quantification of degradation products from ceramics”) since DMEM/F12-10% FBS reproduces more accurately the actual composition of the intercellular fluid, and, furthermore, will lead to a homogenous cross-interpretation of data inferred by the different employed biological assessments.

Amounts (0.1 g) of pure HA and HA substituted with Ce, Mg, Sr, and Zn NPs were added to 2 mL of DMEM/F12-10% FBS and then vortexed for 5 s. The specimens were incubated for 48 h, under agitation, in homeostatic conditions (i.e., humid atmosphere, 37 °C, 5% CO_2_). The powders were separated via centrifugation and filtration. The medium was further diluted by a factor of 100 with ultra-pure milli-Q water to minimise the nonspectral interferences and plasma instability arising from the abundant organic components in DMEM/F12-10% FBS. The ionic concentrations (expressed in ppm = mg/L) were determined by analysing each diluted testing medium via the inductively coupled plasma mass spectroscopy (ICP-MS) technique using a PerkinElmer ELAN DRC-e ICP-MS quadrupole-based (PerkinElmer Corporation, Waltham, MA, USA) device. The measurements were performed in triplicate and the data will be presented as arithmetic mean ± standard deviation.

#### 2.3.2. Cell Culture and Cell Treatments

Murine fibroblasts NIH/3T3 (ATCC^®^ CRL−1658™) and human endothelial EAhy 926 (ATCC^®^ CRL-2922™) cell lines were routinely cultivated in 5% CO_2_ atmosphere and 37 °C in recommended cell media supplemented with 10% FBS and 1% antibiotic. Human osteoblasts hFOB 1.19 (ATCC^®^ CRL-11372) were grown, as recommended, at 34 °C. The NIH/3T3 cell line was used for preliminary screening of cytotoxicity, whereas the human cell lines were employed for the assessment of biofunctional effects of HA-based NPs.

#### 2.3.3. Cell Culture Biological Assessments

The in vitro cytocompatibility experiments of the pure and substituted HA NPs were performed in accordance with the specifications of the ISO 10993-5:2009 standard: “Biological evaluation of medical devices—Part 5: Tests for in vitro cytotoxicity”.

*Sample preparation.* HA-based NPs (0.25 g), with and without cation substitution, were re-suspended in phosphate-buffered saline (PBS) without Ca^2+^/Mg^2+^ with a final volume of 5 mL, resulting in a primary stock of 50 mg/mL NPs, which was stored at −20 °C. This stock was further diluted in a complete cell medium 48 h prior to cell treatments, vigorously vortexed, and incubated at 37 °C for 48 h in the incubator.

Since our main ultimate goal will be the fabrication of cation-substituted HA bone graft substitutes (i.e., porous scaffolds), followed by an extensive assessment from mechanical and biological standpoints, exchange surface equalisation calculations were made (taking into account the size; surface area and mass of NP; geometry and surface area of scaffolds) to attain congruent dissolution processes and, consequently, to subject the cells to similar conditions in both NP and printed porous scaffold form cases. The following constants were considered: cubical scaffold with a side of 10 mm, extruder nozzle diameter of 250 µm, distance between printed “rods” of 250 µm, cell culture medium of 2 mL, and a ~30 nm average NP diameter. An HA NP concentration of 50 µg/mL resulted.

*Cell viability and cell death assays*. The viability of the NIH/3T3 cell line was evaluated by the standard MTS (3-(4,5-dimethyl thiazol-2-yl)5-(3-carboxymethoxyphenyl)-2-(4-sulfophenyl)-2H-tetrazolium) cell proliferation test (Promega Corporation, Madison, WI, USA). Ten thousand cells were incubated in 96-well plates, left to adhere overnight and treated the next day with NPs at a dose of 50 µg/mL for 48 h. Fifty microlitres of cell medium was harvested for the subsequent lactate dehydrogenase (LDH) release assay and the rest of the medium was discarded. The cells were further incubated for at least 1 h with 120 µL of MTS reagent in the completed cell medium (1:5 vol/vol) and the absorbance was read at 490 nm using a Zenyth 3100 (Anthos Labtec Instruments, Salzburg, Austria) multimode reader. The cytotoxicity of the analysed specimens was evaluated by the quantification of LDH release in the culture medium using the CytoTox 96^®^ Non-Radioactive Cytotoxicity Assay (Promega Corporation, Madison, WI, USA). After 48 h of cell incubation without HA NPs, 50 µL of supernatant was harvested and transferred into 96 micro-well plates. Further, 50 µL of LDH substrate solution, according to the producer‘s specification, was added to each well and the plates were incubated for 30 min in dark at room temperature. The reaction was stopped by the addition of 50 µL of stop buffer, and the absorbance was read at 490 nm with 620 nm reference using the same Zenyth 3100 multimode reader. The optical density (OD) of each sample was determined by subtracting the 620 nm values from the 490 nm ones and further corrected for background (cell-free complete medium without HA NPs). The results were expressed as the arithmetic mean ± standard deviation of the OD triplicates.

*Real-time impedance readings.* The cell adhesion and cell proliferation were assessed using the xCELLigence RTCA dual purpose platform (Agilent, Santa Clara, CA, USA). For cell adhesion, E-16 plates were incubated for 1 h with 100 µL complete cell medium, with and without HA NPs solutions, for background readings. Ten thousand cells in 100 µL were added per well and the impedance was recorded every 5 min for 2–4 h. For cell proliferation, E-16 plates were incubated for 1 h with 100 µL complete cell medium, no HA NPs solutions, for background reading, then 40,000 cells in 100 µL were added per well. The cells were left to adhere and proliferate for 24 h until a plateau was reached, then the HA NP solutions were added to the respective wells. To calculate the cell index, normalisation was performed just before addition of HA NP solutions. The impedance readings were exported using the xCELLigence software platform (Agilent, Santa Clara, CA, USA). Doubling times were calculated using the same xCELLigence software.

#### 2.3.4. Statistical Analysis

The cell responses obtained for the SHAs were compared to the pure HA by estimating the *p*-value via a one-way ANOVA multiple analysis comparison followed by a Dunnett’s post hoc test using the statistical analysis GraphPad Prism v.7 software (GraphPad Software, San Diego, CA, USA).

## 3. Results

### 3.1. Composition Analysis via EDXS

**Figure 1:**
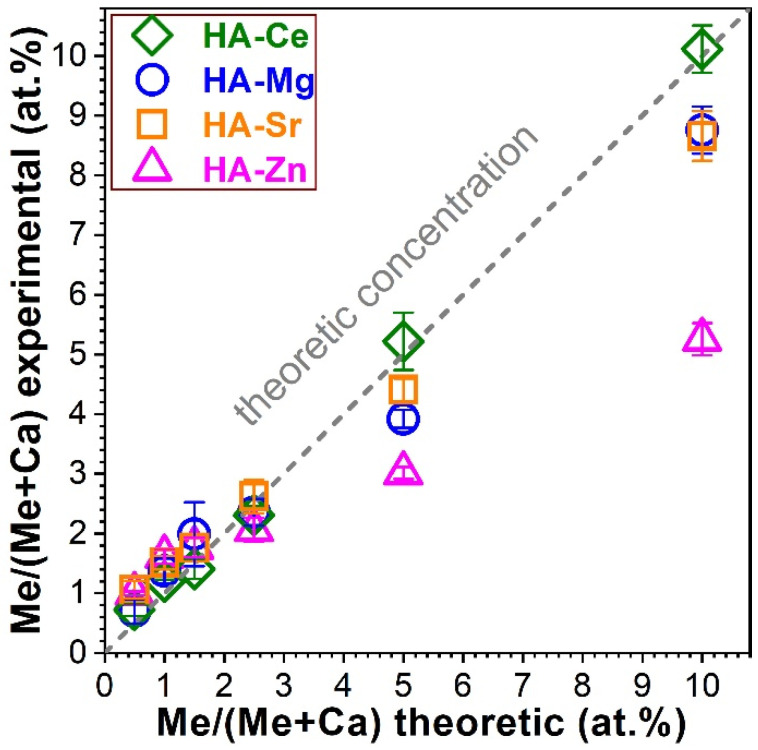
Experimental substituent (Me) concentrations reported to the Ca content, as determined on the basis of quantitative energy dispersive X-ray spectroscopy analyses for the hydroxyapatite (HA)-based nanopowders (NPs). The experimental data are presented as means ± standard deviations (n = 4). The theoretical/nominal substituent values are represented by the dashed line.

The EDXS inferred dopant concentrations, defined as (Me/(Me + Ca) (at.%)) (where, Me = Ce, Mg, Sr, and Zn), are presented in Figure 1 with respect to the nominal/intended reference values. A fairly good replication of the theoretical concentration of the dopants was achieved in the case of Ce, Sr, Mg, and (partially) Zn. Noteworthy experimental vs. theoretical deviations were encountered for the HA with highest concentration of Zn (i.e., 5 at.%). The experimental concentration of Zn was almost half of the intended one. Following these observations, we decided to also synthesise SHA powders with 10 at.% nominal cation substituent concentration. The EDXS data (Figure 1) strengthened the previously observed trends, showing that while Ce, Mg, and Sr can be still well-incorporated into HA (even at these significantly higher contents), in the case of Zn only half of the intended concentration was introduced. Consequently, to enable a more relevant comparison, Zn-substituted HA with the experimental doping concentration value close to the nominal threshold of 5 at.% was further used.

### 3.2. Chemical State Analysis of Cerium by XPS

**Figure 2:**
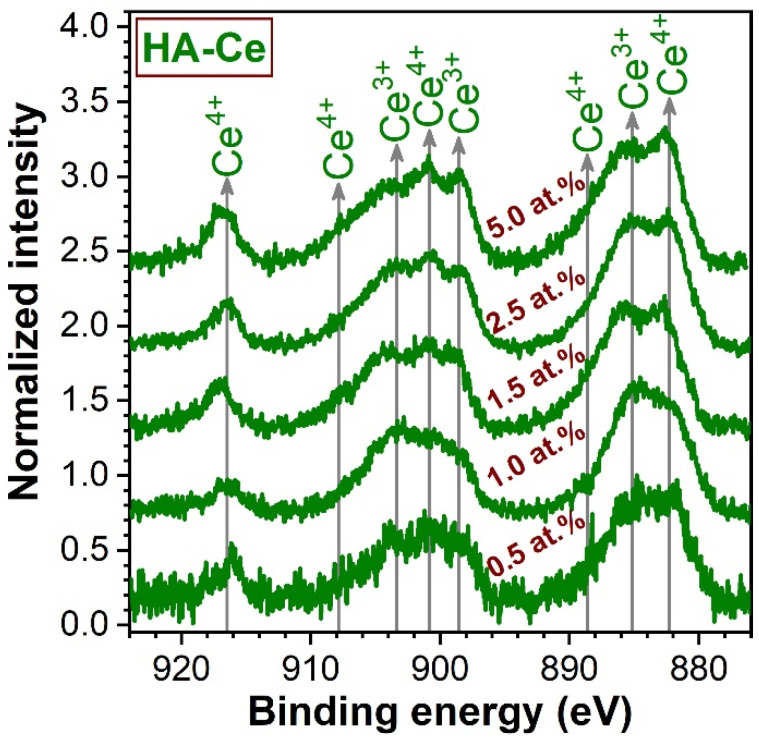
X-ray photoelectron spectra collected in the Ce 3d core electron level region in the case of HA-Ce NPs.

To the difference of the divalent Mg, Sr, and Zn cations, Ce ions can present different valence states (i.e., 3+ and 4+). XPS analyses were carried-out on Ce-HA to infer the valence state of the incorporated Ce (Figure 2). Eight peaks were evidenced and were assigned according to the convention established by Burroughs et al. [58]. The Ce 3d core electron level spectra indicate that Ce^3+^ and Ce^4+^ coexist in HA. This was found to be in good agreement with the scientific literature [37]. Both Ce^3+^ and Ce^4+^ have higher positive charge than Ca^2+^ and therefore Ce may substitute Ca by the formation of vacancies [37]. The Ce^3+^/Ce^4+^ ratio seemingly decreased with the increase of Ce concentration in HA.

### 3.3. Morphological Investigation

**Figure 3:**
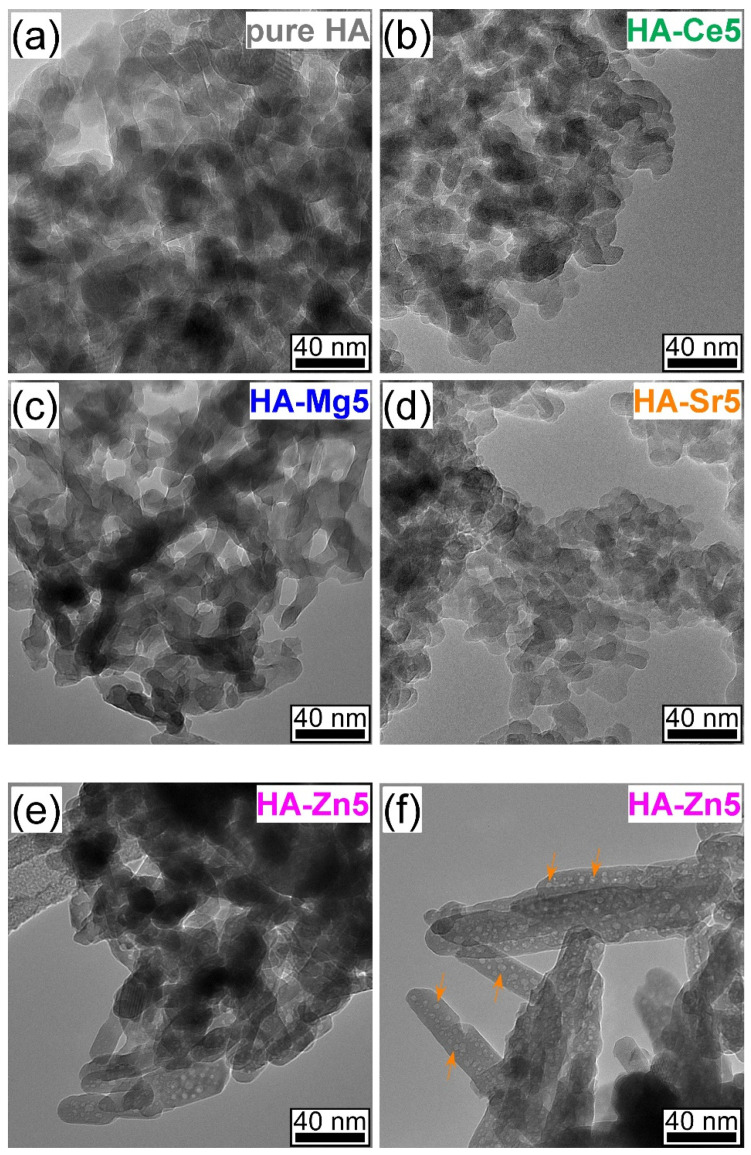
Characteristic comparative bright field transmission electron microscopy images of (**a**) pure HA and 5 at.% (**b**) Ce, (**c**) Mg, (**d**) Sr, and (**e**,**f**) Zn-substituted HA.

The TEM images unveiled that both the pure and substituted HA NPs consisted of oval-shaped nanograins with various degrees of ellipticity/oblateness. The grain size of pure HA, as determined by the ImageJ open source dedicated software (National Institutes of Health, Bethesda, MD, USA), was found to be distributed in the range 12–50 nm with an average of 27 ± 10 nm. This is in good agreement with the values typically reported in the literature [59]. Marked morphological changes were noticed at the highest cation-substituent concentration (Figure 3a–f), with the grain size becoming predictably lower as a consequence of the structural alterations that the doping produces on the HA lattice [6,38,59,60,61]. Due to their reduced sizes, the SHA NPs have a tendency to agglomerate. Interestingly, consistently lower grains were noticed in the case of the HA-Sr5 (Figure 3d). Currently, it is believed that the changes induced in HA by a massive Sr substitution are linked to an increase of the density of grain boundaries and triple junctions, which in turn could enhance the dissolution rate in physiological media [61]. Another peculiarity was noticed in the case of HA-Zn5, which consisted of two morphologically distinct types of NPs (Figure 3e,f). A part of the HA-Zn5 NPs was morphologically similar to the ones met in the case the other HA-based materials, being found quite agglomerated (less dispersed) (Figure 3e). The other part of the HA-Zn5 nanoparticles was elongated with the long axis reaching lengths of 180 nm and with the short axis ~20–35 nm. This latter type of NPs was seemingly better dispersed but featured a high density of round cavities (Figure 3f) with radii of 5–7 nm (a few of them are marked with orange arrows in Figure 3f for easy identification).

### 3.4. Structural Characterisation

#### 3.4.1. FTIR Spectroscopy

**Figure 4:**
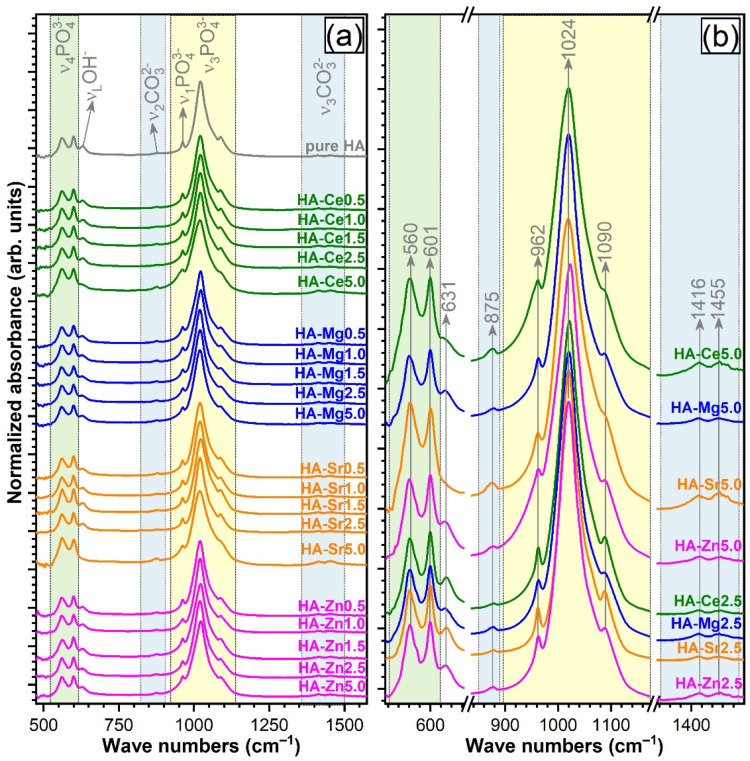
(**a**) Fourier-transform infrared spectra in attenuated total reflectance mode (FTIR-ATR) of all pure and substituted HA (0.5 to 5 at.%). (**b**) Comparison of FTIR-ATR spectra for HAs with substituent concentration of 2.5 and 5 at.%.

Figure 4a shows the FTIR-ATR spectra recorded for both pure and single-substituted HA NPs. All the typical IR absorption bands characteristic to vibration modes of orthophosphate (PO_4_^3−^) groups in an HA compound were evidenced [62,63]: triply degenerated ν_4_ bending (~560 and 600 cm^−1^), nondegenerated ν_1_ symmetrical stretching (~962 cm^−1^), and triply degenerated ν_3_ asymmetric stretching (~1020 and 1090 cm^−1^). The band at ~631 cm^−1^, attributed to ν_L_ libration mode, indicates the presence of hydroxyl groups (OH^–^) in the HA structure. Furthermore, typical vibrations of carbonate groups were revealed for all HA NPs: ν_2_ stretching vibrations of (CO_3_)^2−^ (~875 cm^−1^) and ν_3_ asymmetric stretching of (CO_3_)^2−^ (~1420 and 1451 cm^−1^). The position of these bands suggested a B-type carbonate substitution (i.e., in the lattice sites of (PO_4_)^3−^) [6,62,64]. One should stress that the main bone mineral component is actually a carbonated HA (primarily B-type [65]), substituted with microelements.

Up to the theoretical substitution level of 2.5 at.% (fairly well-replicated experimentally), all SHA NPs had similar FTIR spectra profiles with respect to the pure HA (Figure 4a,b), denoting analogous short-range order. Important differences were recorded in the case of the 5 at.%. cation-substitution level. Whereas for Zn- and Mg-substituted HAs the allure of the FTIR-ATR spectra was conserved from 2.5 to 5 at.%, in the case of Ce and even more for Sr, a broadening of the spectral envelopes and less conspicuous ~1090 cm^−1^ peaks were noticed (Figure 4a). This indicates that a certain degree of structural (short-range) disorder is inflicted by the incorporation of the two cations at such a concentration level. The results are in agreement with the TEM observations, which pointed out that HA-Ce5, and especially HA-Sr5, presented the lowest grain sizes.

Interestingly, the broadening of the FTIR spectra of HA-Ce5 and HA-Sr5 was accompanied by the reduction (for HA-Ce5) or even extinction (for HA-Sr5) of the ν_L_ libration mode band of structural hydroxyl groups (~631 cm^−1^) and the increase of the ν_2_ (~875 cm^−1^) and ν_3_ stretching (~1420 and 1451 cm^−1^) vibration bands of carbonate units, which will be discussed later.

#### 3.4.2. Micro-Raman Spectroscopy

**Figure 5:**
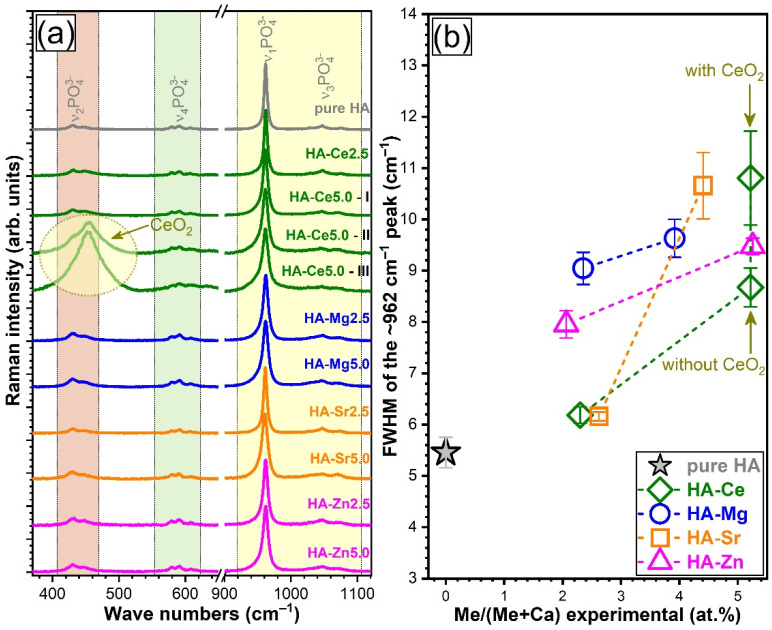
(**a**) Micro-Raman spectra for the pure and substituted HAs (2.5 and 5 at.%), collected in the wave number range 370–1120 cm^−1^ and the (**b**) corresponding full-width at half maximum (FWHM) values of the ν_1_ symmetric stretching mode band (~962 cm^−1^).

Complementary to FTIR spectroscopy, micro-Raman spectroscopy measurements were carried for the pure and highly substituted (i.e., 2.5 and 5 at.%) HA samples (Figure 5a). All Raman spectra were characteristically dominated by the ν_1_ symmetric stretching mode band (at ~962 cm^−1^) of orthophosphate groups in an HA-type lattice [66,67,68,69]. The lower intensity maxima centred at ~430, ~580−610, and ~1030−1080 cm^−1^ appertain to the doubly degenerated ν_2_ bending, triply degenerated ν_4_ bending, and triply degenerated ν_3_ asymmetric stretching modes, respectively [66,67,68,69]. Occasionally, depending on the analysed sample region, dissimilar spectral envelopes were unveiled in the low wave numbers region (i.e., 380–530 cm^−1^) of the HA-Ce5-type samples (Figure 5a). Spectra collected on three such different regions (I, II, and III) are presented in Figure 5a. The supplemental maximum at ~455 cm^−1^ exposed the presence of a CeO_2_-like phase at high cation substituent concentration. This was not the case with the other substituted HA NPs, irrespective of the type or content of cation substituent. This is linked to the reduced sampling volume (i.e., ~1.7 µm^3^) of the Raman spectroscopy technique with respect to FTIR spectroscopy or XRD methods, which provide a rather global sample information. The results suggest that the secondary CeO_2_-like phase is found randomly dispersed/amassed at a micrometre level within the NPs.

The full-width at half maximum (FWHM) of the intense band positioned at ~962 cm^−1^ enlarged with the substituent concentration from 2.5 to 5 at.% (Figure 5b), irrespective of the type of substituent. A significantly larger increase was noticed for the Sr-HAs (Figure 5b). This is indicative of a more substantial lattice distortion upon Sr incorporation. Large FWHM values were also recorded in the case of the Ce-HA with the highest substituent concentration when the secondary CeO_2_ phase was detected in the spectrum. The lesser distortions were observed for the Mg- and Zn-substituted HAs.

#### 3.4.3. X-Ray Diffraction

##### Qualitative Assessment

**Figure 6:**
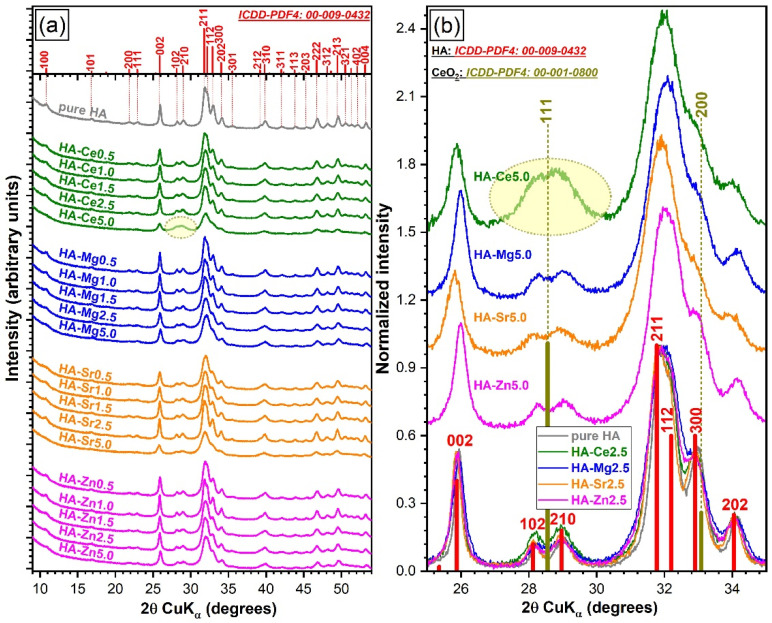
(**a**) X-ray diffraction patterns of all pure and substituted HAs (0.5 to 5 at.%). (**b**) Comparison of XRD patterns for HAs with substituent concentrations of 2.5 and 5 at.%, zoomed in the 2θ angular region ≈ 24–35°. The reference diffraction files of hexagonal hydroxylapatite (ICDD: 00-009-0432) and/or cubic CeO_2_ (ICDD: 00-001-0800) are represented as bars.

The XRD patterns of the pure and substituted HA powders are represented comparatively in Figure 6a. All samples, with the exception of HA-Ce5, exclusively consisted (at the sensitivity limit of the employed analysis equipment) of a hexagonal HA phase (ICDD: 00-009-0432, space group P63/m (176)). Up to a dopant concentration of 1.5 at.%, the XRD patterns of all SHAs elicited seemingly unchanged well-resolved, fairly broad (indicative of a nanocrystalline material) maxima, which advocated for a preservation of the structural features. The first discernible structural modifications were noticed in the cases of HA-Mg2.5 and HA-Sr2.5 NPs. HA-Mg2.5 and HA-Sr2.5 NPs presented broader/less-resolved and sharper/better-resolved (similar to the ones of pure HA) diffraction maxima (Figure 6a), respectively, with respect to the corresponding SHA NPs with cation substitution levels in the range 0.5–1.5 at.%.

For a substituent concentration of ~5 at.%, the diffraction peaks of all SHAs decreased in intensity (markedly for the HA-Ce5 and HA-Sr5 NPs) and became clearly wider and ill-resolved. Both are “symptoms” of an increase of the structural long-range disorder. In the case of the HA-Ce5 NPs, the unusual high intensity of the HA 102 and 210 diffraction peaks region (with respect to the relative intensity of corresponding peaks in the reference ICDD file) suggested the superimposition of the dominant diffraction maximum (i.e., 111) of a secondary minor phase, identified as CeO_2_ (ICDD: 00-001-0800) (Figure 6b) and later confirmed via Rietveld analysis to have a share of ~10 wt.%. The CeO_2_ formation might occur at an even lower dopant concentration, but if so, the content of such a secondary phase would be situated below the sensibility limit of the employed XRD apparatus.

In the light of these findings, we decided to further focus our research (namely, the quantitative structural investigations and biological assessments) only on the NPs with the lowest, intermediate (i.e., ~2.5 at.%, where structural modifications debuted), and highest substituent concentration.

##### Quantitative Assessment of Crystalline Quality

The above-described qualitative structural trends were supported by the quantitative assessments performed by the determination of the (i) average crystallite size along the *c*-axis and (ii) crystallinity index (derived from the FTIR spectra). The latter gauge, even though not often employed, has been found to be an excellent tool for the reliable valuation of the HA crystallinity [70]. The crystallinity index (CI) was calculated by the summation of the heights of the absorption peaks at ~600 and 562 cm^−1^ (after baseline subtraction), divided by the height of the minimum situated between them.

**Figure 7:**
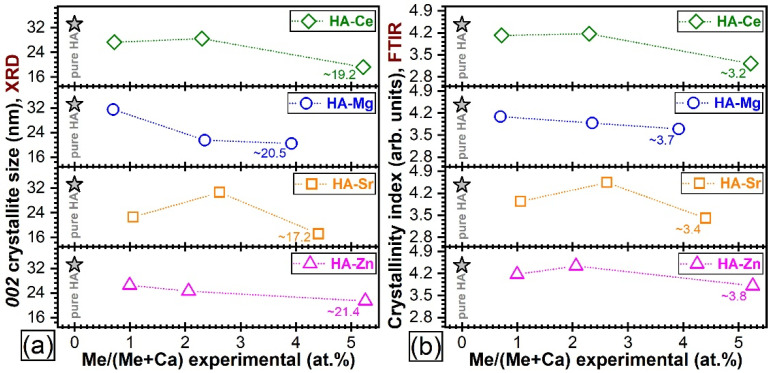
Evolution of the (**a**) crystallite size (as determined on the basis of the Scherrer equation applied to the 002 XRD peak) and (**b**) index of crystallinity (as determined on the basis of FTIR-ATR spectra) with the increase of the substituent concentration. The values inferred in the case of pure HA NPs are presented for comparison.

A marked modification of 002 crystallite size (crystalline coherence length along the *c*-axis) was noticed in the case of Mg^2+^ (i.e., a decrease) and Sr^2+^ (i.e., an increase) substitutions at a concentration level close to ~2.5 at.% (Figure 7a). This was also fairly well-supported by the evolution of the CI values (Figure 7b). Less evident changes were noticed in the case of Ce and Zn dopants at similar intermediate concentrations. Likewise, in the case of highest-used substituent concentration, the FTIR-ATR spectroscopy (Figure 4) and XRD (Figure 6) naked-eye observations were found to be in full agreement with the mean crystallite size and CI values. Explicitly, the lowest crystallite size and CI values were recorded in the case of the HA-Ce and HA-Sr NPs (Figure 7a,b).

##### Variation of Cation Occupation with Substituent Concentration

**Figure 8:**
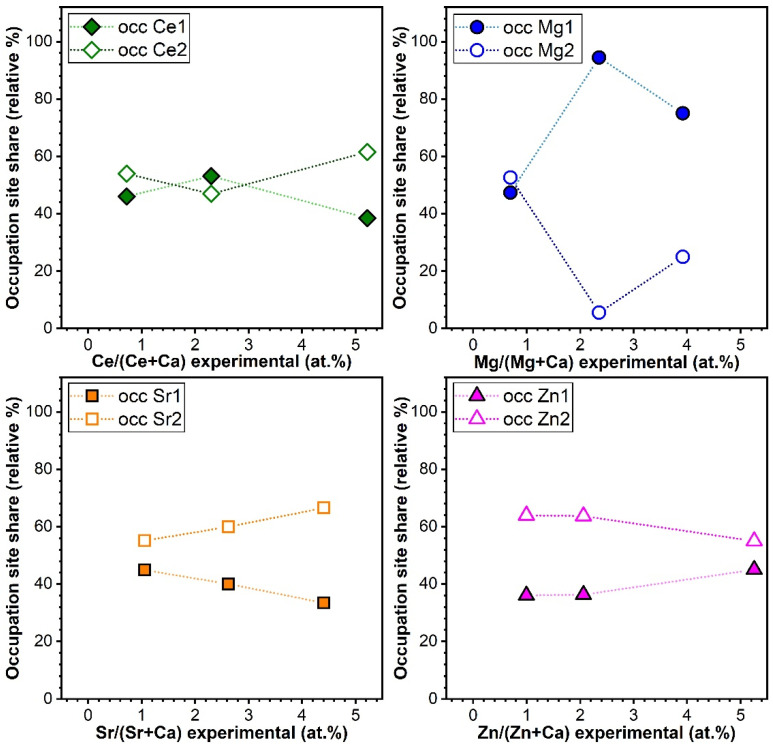
Cation occupation either on Ca(I) (occ**Me**1) or Ca(II) (occ**Me**2) sites as a function of the substituted cation concentration incorporated into the HA lattice.

The Rietveld refinement analysis of the XRD patterns suggested that Sr can occupy the Ca(I) (e.g., occSr1) and Ca(II) (e.g., occSr2) sites in similar shares when in low concentrations. A preferential occupation for the Ca(II) site was recorded for Sr concentrations larger than 2.5 at.% (Figure 8). Ce adopted similar occupation sites to Sr in HA, with a preference for the Ca(II) occupation site at the largest concentration. Furthermore, it was revealed that despite their almost equal ionic radius, Zn prefers to occupy the larger site (II), while Mg the smaller site (I) (Figure 8). This result is in good agreement with the determinations made by Tang et al. [6,71] and Ren et al. [6,72].

##### Modification of the Lattice Parameters

**Figure 9:**
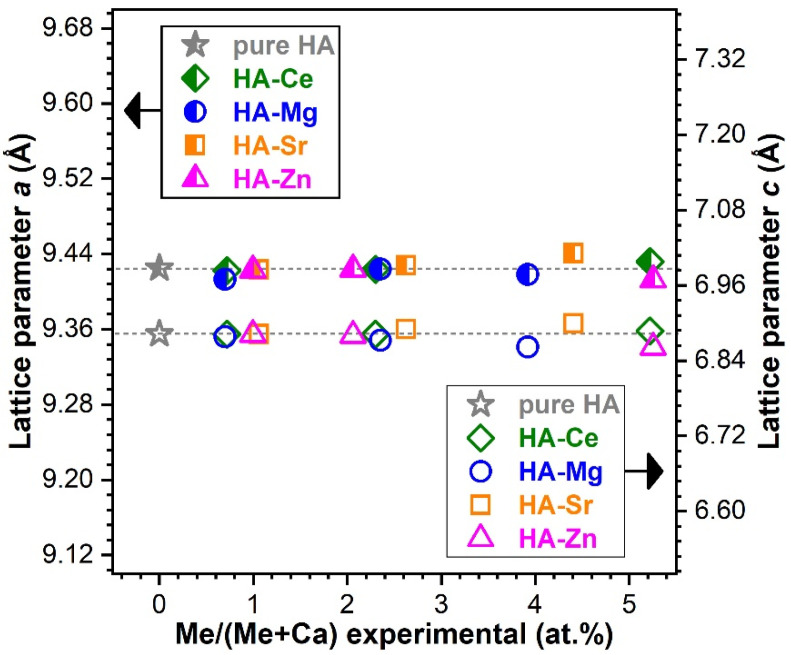
Variation of the *a* and *c* lattice parameters as a function of the cation substituent concentration. The errors (bars) of the lattice parameters (as determined by fitting) were not figured since they were too small to be well-visualised.

Figure 9 presents the modification of the HA lattice parameters as a consequence of the cation substitution (as inferred by the Rietveld structural refinement of the XRD diagrams). HA crystallises in the hexagonal system (space group P63/m (176)) with two formula units per unit cell, each with 44 atoms. The *a* and *c* lattice parameters of pure HA were found to be of 9.4250 and 6.8826 Å, respectively, thus in good agreement with the values obtained in the scientific literature [1,73].

It safe to say that unambiguous lattice parameter modification can be only pinpointed at higher substituent concentrations. The electronegativity of metallic Ce (~1.12) and ionic radius of Ce^3+^ (~101 pm) and Ce^4+^ (~87 pm) are close to those of Ca^2+^ (1.00 and ~100 pm) for a coordination number of 6 [74,75], favouring their easy incorporation into the HA lattice [6]. As expected, there was no noteworthy lattice parameter deviation up to a concentration of ~2.5 at.% with respect to pure HA. As reported in the literature, the smaller ionic radius of Mg^2+^ (~72 pm) and Zn^2+^ (~74 pm) [74,75] generally induces a decrease of the lattice parameters and unit cell volume [76]. The observed changes in lattice parameters for the highest employed concentrations of Zn and Mg were in good agreement with this general expected trend. The *a* & *c* lattice constants of HA were situated at values of 9.4126 & 6.8612 Å and of 9.4182 & 6.8616 Å for the highly doped HA-Zn and HA-Mg, respectively.

For a Sr substituent content of ~5 at.%, both *a* and *c* lattice parameters of HA were perceptibly increased (i.e., *a* ≈ 9.441 Å, *c* ≈ 6.899 Å at ~5 at.%), which was to be expected due to the higher ionic radius of Sr^2+^ (~118 pm) [74,75] in comparison to Ca^2+^.

### 3.5. Biological Assays

#### 3.5.1. Dopant Release Determinations

The reliability of the specimen preparation protocol has been confirmed by the analysis of the testing medium without sample. The ion concentrations of the prominent electrolytes of DMEM-F12 (except the abundant Na^+^ and Cl^−^ ones) as determined by ICP-MS were found to be in good agreement with the product information provided by the cell culture manufacturer [77]. Namely, Ca^2+^, Mg^2+^, K^+^, and P^5+^, having expected concentrations of ~42.1, ~17.3, ~163.5, and ~29.5 ppm, respectively, were found by ICP-MS in amounts of ~46.2 ± 4.6, ~21.3 ± 0.8, ~136.5 ± 7.8, and ~31.9 ± 1.9 ppm, respectively.

**Figure 10:**
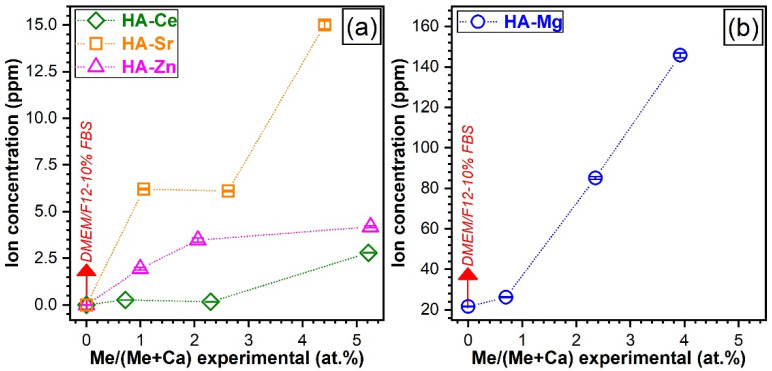
The concentrations of the (**a**) Ce, Sr, Zn and (**b**) Mg dopants leached after 48 h in the DMEM/F12-10%FBS solution by the SHA NPs, as determined by inductively coupled plasma mass spectrometry. The experimental data are presented as means ± standard deviations (n = 3).

The dopant concentrations released by the SHA NPs in the DMEM/F12 culture medium (Sigma D6421) supplemented with 10% FBS, after 48 h of incubation, as assessed by ICP-MS measurements are presented in Figure 10.

A first important observation is that Ce was released in extremely low contents (0.17–0.27 ppm) up to a substitution level of ~2.5 at.%. Only for the highest doped Ce-HA a more perceptible Ce ion release (of ~2.8 ppm) was evidenced (Figure 10a). This contrasted with the trend recorded for Mg-HAs. Mg was released in a dose-dependent manner, eliciting a quasilinear evolution with the substituent concentration (Figure 10b). Its faster release compared to other elements was conspicuous, reaching a maximum value of ~124 ppm (after the deduction of Mg experimental concentration of the testing medium), which was ~44 times higher with respect to Ce-HA. Sr and Zn are also released in a dose-dependent manner, with the notable exception of HA-Sr2.5, which presented a similar leached Sr concentration to HA-Sr0.5. This can be associated with the improved crystalline quality observed for the HA-Sr2.5 (see Figure 6a and Figure 7a).

Since the ion release in complex organic–inorganic testing media (such as the DMEM-based ones) are a relatively new field of research, more systematic and insightful investigations are needed and are envisaged in the near future to elucidate beyond doubt the ion release mechanisms at hand. This way, tools to tune the release of therapeutic ions, adapted to specific illnesses and patients, might be devised.

The incorporation of therapeutic ions into HA NPs could be of great bio-functional interest due to their ability to improve the osteogenesis and/or angiogenesis capability of the bioceramics and allow for the generation of a thriving cellular environment. To that aim, the effect of the SHA NPs on adhesion, proliferation, and viability of fibroblast, osteoblast, and endothelial cells lines was further explored.

#### 3.5.2. Cytotoxic Evaluation with Standard Colorimetric Assays

**Figure 11:**
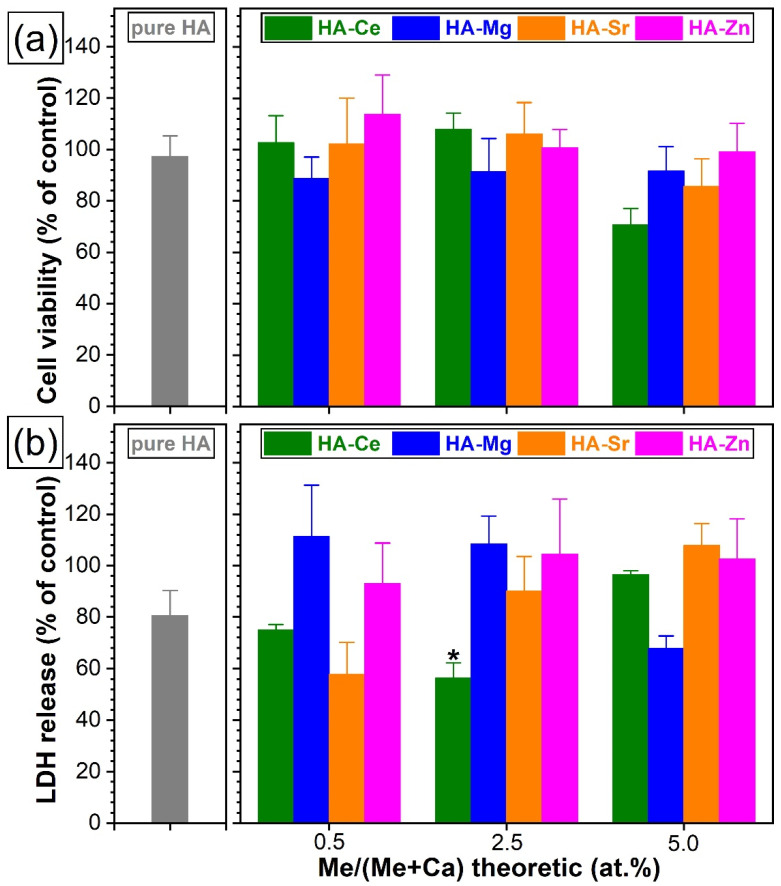
The NIH/3T3 (ATCC^®^ CRL−1658™) mouse fibroblast (**a**) cell viability (evaluated by a 3-(4,5-dimethyl thiazol-2-yl) 5-(3-carboxymethoxyphenyl)-2-(4-sulfophenyl)-2H-tetrazolium assay—MTS assay) and (**b**) cytotoxicity (inferred by a lactate dehydrogenase release test—LDH) evaluated after 48 h in the case of the pure and substituted HA NPs. The (**a**) MTS and (**b**) LDH results are presented as means ± standard deviations (n = 3) and were obtained as a percentage of the control. * *p* < 0.05, statistically significant differences with respect to pure HA as determined by using a one-way ANOVA multiple analysis followed by a Dunnett’s post hoc test.

The in vitro evaluations debuted with a cytotoxicity screening test (performed in accordance to ISO 10993-5:2009 recommendations) on a mouse fibroblast cell line (NIH/3T3, ATCC^®^ CRL−1658™). The cell viability and death were assessed by MTS and LDH tests, respectively (see Figure 11). The pure HA NPs presented a cell viability response comparable to the one recorded for the biological control (i.e., the cell culture medium without sample). The SHA NPs elicited similar cell viability results as compared to the pure HA (without statistically significant differences, *p* > 0.05), with one exception, the SHA NPs with the highest content of Ce (*p* < 0.05) (Figure 11a). The reduction in cell viability can be ascribed to the presence of the secondary nano-sized CeO_2_ phase (Figure 5 and Figure 6) [78]. A lower LDH release was signalled in the case of pure HA with respect to the control (Figure 11b). The LDH data further indicates that HA NPs substituted with Ce, Mg, Sr, or Zn showed no statistically significant differences (*p* > 0.05) with respect to pure HA. Only in the case of HA-Ce2.5 a slight inhibition of the LDH activity was noticed (*p* < 0.05). Consequently, it was decided to further evaluate the biofunctional response of all these type of HA NPs (without any exclusion) with respect to two relevant human (i.e., osteoblast hFOB 1.19 (ATCC^®^ CRL-11372) and endothelial EAhy 926 (ATCC^®^ CRL-2922™)) cell lines.

#### 3.5.3. Measurement of Cell Adhesion and Proliferation via Real-Time Impedance Readings

##### Assessment of Osteoblast (hFOB) and Endothelial (EAhy) Cell Adhesion via RTCA

**Figure 12:**
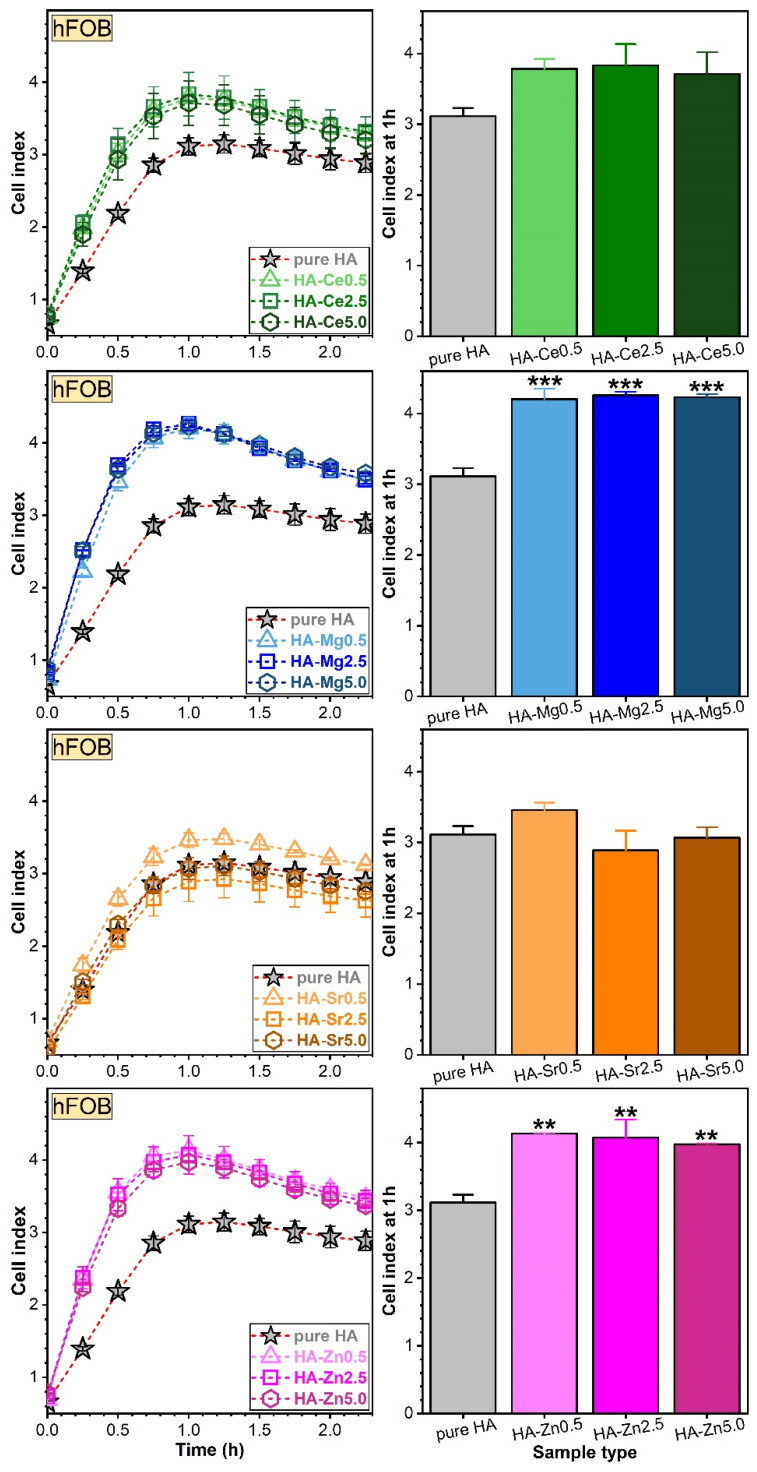
Impedance changes of the real-time cell analysis (RTCA) system mirroring the adhesion of osteoblast (hFOB) cells in the presence of pure HA and Ce-, Mg-, Sr-, or Zn-substituted HA (**left panel**). Cell indices at 1 h were plotted as bars (**right panel**). ** *p* < 0.01 and *** *p* < 0.001 statistically significant differences with respect to pure HA (taken as control), as determined by using a one-way ANOVA multiple analysis followed by a Dunnett’s post hoc test.

**Figure 13:**
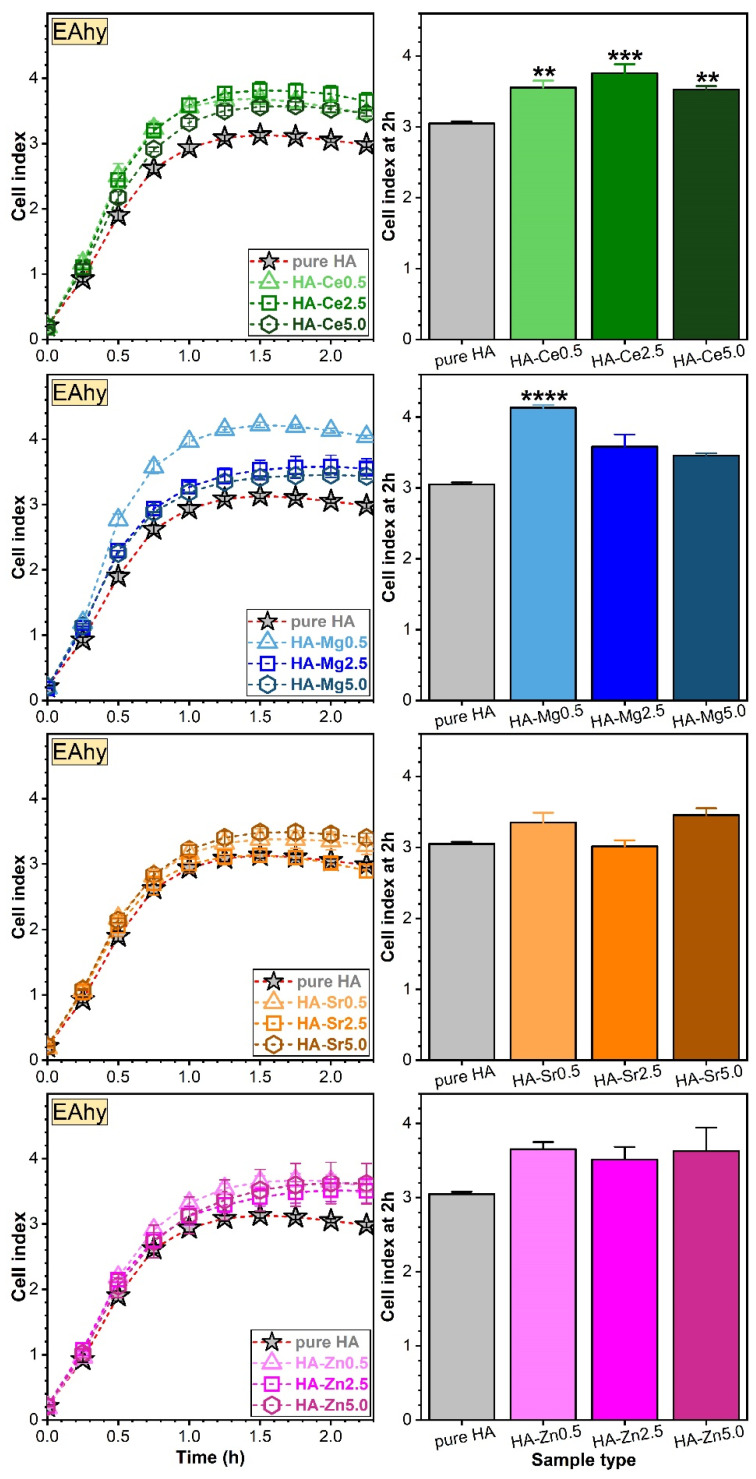
Impedance changes of the RTCA system mirroring the adhesion of endothelial (EAhy) cells in the presence of pure HA and Ce-, Mg-, Sr-, or Zn-substituted HA (**left panel**). Cell indices at 1.5 h were plotted as bars (**right panel**). ** *p* < 0.01, *** *p* < 0.001, and **** *p* < 0.0001 statistically significant differences with respect to pure HA (taken as control), as determined by using a one-way ANOVA multiple analysis followed by a Dunnett’s post hoc test.

hFOB (Figure 12) and EAhy (Figure 13) cells were treated with 50 µg/mL of pure or Ce-, Mg-, Sr-, and Zn-substituted HA and incubated in the RTCA system for at least 3 h. Except for the Sr-HA NPs with Sr contents of ~2.5 and ~5 at.%, all SHA NPs favoured the adhesion of the human osteoblasts when compared to the pure HA (taken as control) (Figure 12, left panel). The cell index depends on the number of cells [79], and for the experiments presented within the value of the cell index was situated between 2 and 4.5. The maximum attachment index of osteoblasts was reached at 1 h of incubation and was further used to infer the statistically significant differences (if any) between samples (Figure 12, right panel). Statistically significant differences in terms of hFOB adhesion were found for the Mg- and Zn-substituted HA NPs with respect to the pure HA.

The adhesion of endothelial cells was stimulated by the low dosage (Ce, Mg, and Zn) cation substitution in HA (Figure 13, left panel). The EAhy cell adhesion values were quite indifferent for the Sr-substituted HA NPs (Figure 13-left panel). The maximum attachment index of endothelial cells, reached at 1.5 h, was further used to assess the statistically significant differences between samples (Figure 13, right panel). The most salient EAhy cell adherence was found to be favoured by the SHA NPs with a Mg content of ~0.7 at.%. Of note, none of the SHA NPs performed poorer than pure HA in terms of cell adhesion. Thus, for any chosen SHA the efficiency of cell adhesion would be at least equal to the one of pure HA. In order to further investigate potential benefits, the impact of cation substitution on cell proliferation was addressed.

##### Assessment of Osteoblast (hFOB) and Endothelial (EAhy) Cell Proliferations via RTCA

**Figure 14:**
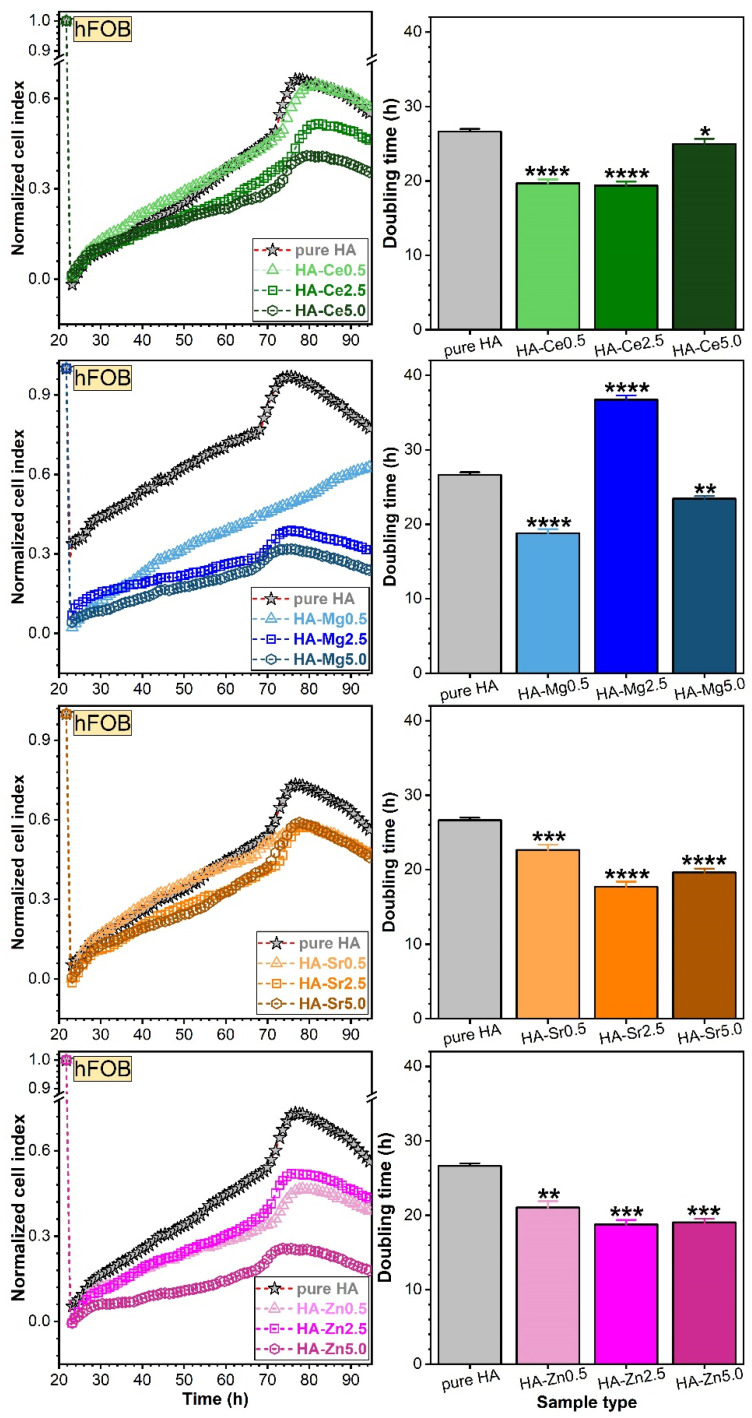
Impedance changes of the RTCA system mirroring the proliferation of hFOB cells in the presence of pure HA and Ce-, Mg-, Sr-, or Zn-substituted HA (**left panel**). The error bars were not figured as they would have obstructed the good visualisation of the evolution of mean values. The values of the doubling time were plotted as bars (**right panel**). * *p* < 0.05, ** *p* < 0.01, *** *p* < 0.001, and **** *p* < 0.0001 statistically significant differences with respect to pure HA (taken as control), as determined by using a one-way ANOVA multiple analysis followed by a Dunnett’s post hoc test.

**Figure 15:**
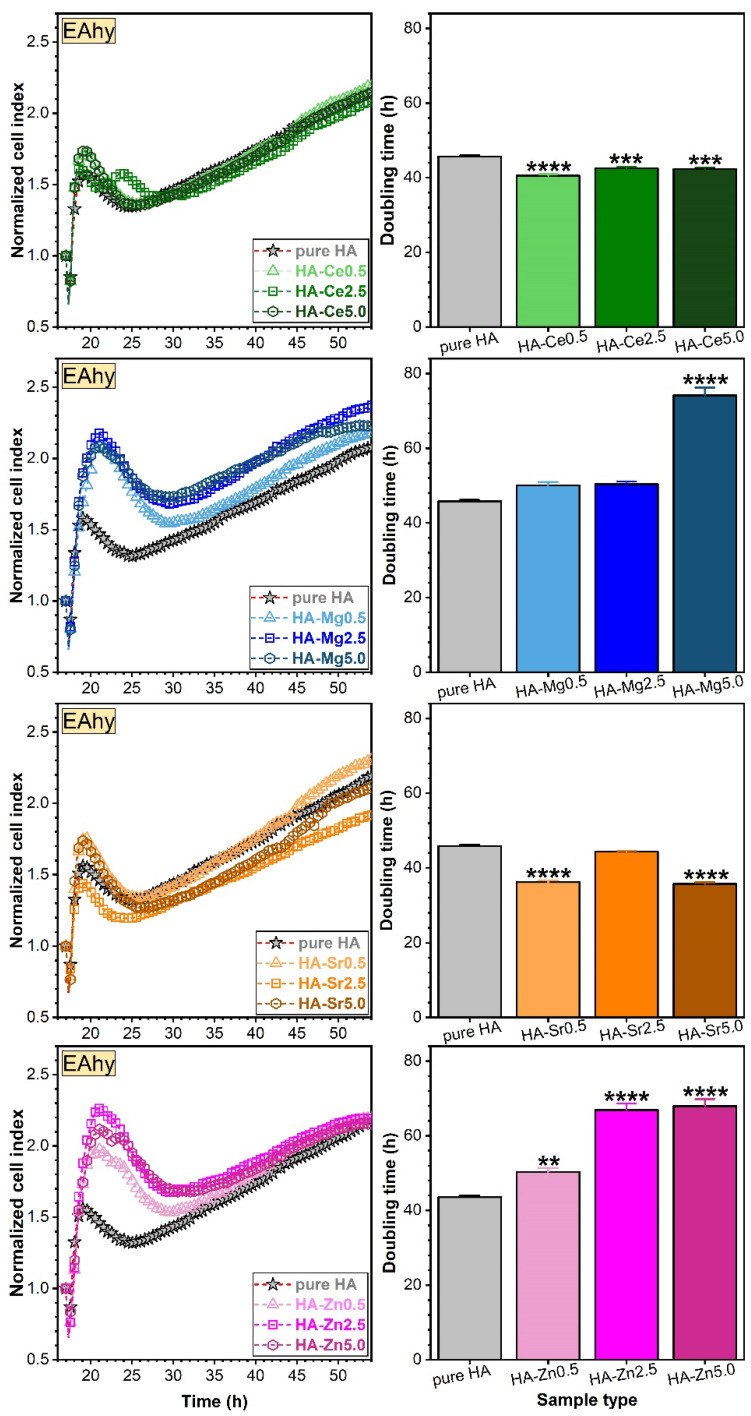
Impedance changes of the RTCA system mirroring the proliferation of EAhy cells in the presence of pure HA and Ce-, Mg-, Sr-, or Zn-substituted HA (**left panel**). The error bars were not figured as they would have obstructed the good visualisation of the evolution of mean values. The values of the doubling time were plotted as bars (**right panel**). ** *p* < 0.01, *** *p* < 0.001, and **** *p* < 0.0001 statistically significant differences with respect to pure HA (taken as control), as determined by using a one-way ANOVA multiple analysis followed by a Dunnett’s post hoc test.

hFOB (Figure 14) and EAhy (Figure 15) cells were first allowed to adhere and proliferate on the E-16 plate up to a steady state, normalised to cell index 1, and then treated with the pure and substituted HA NPs. Hence, the subsequent modification of the cell index was a direct result of the treatment and was not biased by differences in cell number between different wells.

To assess the long-term impact on cell proliferation, doubling time (DT) was determined, using the xCelligence software. The time interval chosen for calculation of DT included ascending slopes for all cells. The DT is defined as the time taken by the cell population to double in size. Mathematically, this time can be calculated by dividing the natural logarithm of 2 by the rate constant of cell production K_p_ (*T_d_ = ln2/K_p_*) [80,81] and is linked in the RTCA analysis with the cell index (CI) by the proportional relationship *CI = CI*(*time =* 0) × 2^(*time/DT*).

The results indicate a good proliferation capability of EAhy cells for all SHA NPs in comparison with pure HA. The highest cell index values were obtained for Mg- and Zn-substituted HAs (Figure 15). No such exponential growth at an early stage was noticed for the osteoblast cells, pointing out a significant growth difference proficiency between the two cell phenotypes.

In the long-term, all SHA NPs induced improved hFOB cell proliferation responses with respect to pure HA, as indicated by low doubling times (Figure 14). However, this cannot be said for the long-term EAhy cell proliferation values recorded for the Mg- and Zn-substituted HA NPs, which provided suboptimal proliferation in contrast to the Ce- and Sr-substituted ones. The Sr substitution in HA generally favoured the hFOBs (Figure 14) and EAhy (Figure 15) cell proliferation. Correlations were sought between initial cell adherence (Figure 12 and Figure 13) and proliferation values (doubling times) in an attempt to better understand the mechanisms at play. However, the data was rather disparate, and no straightforward association was established for all type of materials and cells. Nevertheless, the materials that led to best proliferation values for both hFOB and EAhy cells were the Sr- and Ce-substituted HA NPs, which did not present the best initial cell adherence.

## 4. Discussion

Currently, the biomedical orthopaedic and dentistry fields are witnessing an ever-growing demand for a large variety and number of bone fillers, bone graft substitutes, and endo-osseous implants, which could improve the quality of life of millions of patients each year (if considering the fewer bone grafts procedures alone [82]). It is therefore no surprise that intensive research is being conducted worldwide to identify bioactive materials capable of stimulating bone repair and tissue regeneration [5,6,7]. Recently, cation-substituted HAs have started to become acknowledged as remarkable candidate materials for bone-related applications (i.e., treatment, reinforcement, or substitution). Designed cationic substitutions in the HA lattice could induce a controlled modification of morphology, lattice parameters, crystalline quality, and surface energy with an effect on the functional mechanical and biological performance (e.g., biomineralisation capacity, surface reactivity/ready adsorption of proteins/growth factors, cell proliferation and differentiation, osteogenic and angiogenic capability, antimicrobial effects) [5,6,7].

Although, in the last period, numerous (but disparate) articles have been published in this field of knowledge (with the vast majority focusing on a single type of substituent), frequent contradictions have been shown with respect to either the (i) role of a dopant on physicochemical features of HA or (ii) the effect of dopant concentration in HA on cells’ adherence and proliferation. This is why our belief was that it could be of paramount importance to collect and cross-examine in a single concomitant study the physico-chemical characteristics and in vitro biological responses (evaluated on the basis of reliable protocols) of some important SHA biomaterials (i.e., series of HA NPs substituted with perhaps the most interesting, biofunctional wise, cations: Ce^3+/^Ce^4+^, Mg^2+^, Sr^2+^, and Zn^2+^). Such an approach might constitute a step in the right direction, i.e., the unambiguous comparative assessment of these materials’ potential to foster future significant developments in the orthopaedic and dentistry fields.

In the framework of this work, the compositional, morphological, structural properties, and biological responses have been comparatively and insightfully studied in a unitary manner in an attempt to delineate definite hints on their effects.

A first noteworthy observation was that from all four type of cation substituents, Zn experienced by far the larger deviations with respect to the theoretical (expected) concentration when used in concentrations in excess of 2.5 at.% (Figure 1). A possible explanation for the decreased Zn content HA is accommodated by the incomplete chemical reaction of the Zn reagent with the Ca and P ones, with the (unreacted) Zn excess being washed away in the filtration step. This hypothesis was confirmed by TEM data (Figure 3e,f), which evidenced HA-Zn NPs with cavities whose origin could be linked to the removal of the unreacted Zn during the post-synthesis steps (i.e., washing and filtering). The difficulty of incorporating Zn into the HA structure has been sporadically suggested in the scientific literature [1].

From a structural point of view, notable structural modifications have only been observed starting with a cation concentration of ~2.5 at.%. The changes induced in the HA structure by Ca^2+^ substitution can be associated with the differences in ionic radius, electronegativity, and effective charge of the substituting cation [6,7]. Such variances can lead to important structural distortions (in both terms of short- and long-range order alterations), as visually observed in the case of both FTIR-ATR and micro-Raman spectra (Figure 4 and Figure 5) and XRD patterns (Figure 6). The congruence between the data yielded by these different investigation techniques should be emphasised.

Generally, irrespective of type of cation, the crystalline quality of the HA was incrementally reduced, although at different paces. A single discontinuity in the crystalline quality decreasing trend emerged in the case of HA-Sr2.5 (confirmed by both the XRD and FTIR-ATR spectroscopy quantitative assessment—Figure 7), which might be perceived at a first glance as unexpected. However, in the case of Sr doping in HA, the scientific literature records numerous contradictions, with this specific cation substituent both decreasing [29,83] and increasing [84,85,86] the crystalline coherence length of HA. One possible explanation resides in the higher ionicity/electropositivity of Sr with respect to both Ca and the other employed cation substituents [85] being thus able, in some circumstances, to form bonds more readily with non-associated oxygen ions without necessarily cleaving already formed Ca–O bonds. At the highest cation substituent concentration (i.e., 5 at.%) a more marked decrease in crystalline quality was noticed in the case of Ce and Sr. This was first hinted by the low intensity/absence of the IR bands appertaining to the structural hydroxyl groups (Figure 4). This spectral modification has been documented in the scientific literature [87,88,89], and it was linked to a strong reduction of the crystallite size and the subsequent distortions and atomic disorder in the HA lattice, which in turn hampers the incorporation of hydroxyl units. An important lattice distortion effect was to be expected in the case of Ce (as it has higher positive charge than Ca^2+^ and therefore would induce the formation of vacancies upon substitution) and Sr (as it was by far the bulkiest among the selected cations). A complementary lattice distortion effect induced by HA’s carbonatation (as highlighted by the FTIR-ATR spectra) and/or presence residual/secondary phases cannot be excluded either. The micro-Raman spectroscopy results (Figure 5) further strengthened the FTIR-ATR spectroscopy assessments, supporting on the one hand the stronger crystalline lattice alterations/disturbances inflicted by the Sr ions upon their complete incorporation, and on the other hand revealing the presence in certain regions of the sample of CeO_2_ as a secondary minor phase in addition to HA. The supplemental presence of CeO_2_ for the HA-Ce5 sample was also suggested by the increased Ce^4+^/Ce^3+^ ratio in the Ce 3d XPS core electron level region photoelectron spectra (Figure 2) and confirmed beyond doubt by the XRD data (Figure 5a). It is thus indicated that Ce has a lower solubility limit in the HA lattice with respect to the other three types of substituents, and upon increase it determines the segregation of CeO_2_ phase.

The structural changes can be related to the position at which the substituent cation enters the HA lattice, specifically, the Ca(I) or Ca(II) sites. The scientific literature records several controversies associated with the substitution sites of Mg, Sr, and Zn [6]. Yet, to the best of our knowledge, the occupation sites of Ce in HA have not been reported. Ca(II) sites have a larger volume than the Ca(I) ones. A simplistic reasoning might lead to the conclusion that a cation with a larger radius than the one of Ca^2+^ (i.e., ~100 pm) should prefer to occupy the site(II) [1,6]. Nevertheless, other factors such as charge of cation, bond strength, and metal–oxygen distance can also influence the selection of the occupancy position [6,90]. For example, the Metal(I)-O has a longer length than Metal(II)-O and might allow larger cations to be accommodated. The experimental results, presented within, further indicate that a straightforward dependency is difficult to isolate and link to a causality factor alone. For instance, at the low concentration (i.e., ~0.5 at.%), Ce, Mg, and Sr were distributed in the two Ca sites in approximately equal proportions. However, at large Sr concentrations (i.e., >2.5 at.%) a preferential occupation for the Ca(II) site was disclosed (Figure 8). This is in good agreement with several reports, which already showed that an inversion of substitution sites from Ca(I) to Ca(II) occurs when the Sr doping is increased beyond 3.5 at.% [6,91]. This was associated with the prevalence of ionic radius size effect at larger substituent concentrations [6]. A similar trend was recorded for Ce as well at the highest concentration. However, different evolutions have been recorded for Zn compared to Mg, although their ionic radii are almost equal. Mg preferred to occupy, as expected, the more confined Ca(I) site. Instead, Zn had a prevalence for the Ca(II)) site. It is suggested that the substitution of Zn in HA is driven by another influential factor than the ionic radius. This different behaviour of Zn and Mg might be attributed to their highly dissimilar electronic structure and electronegativity [92].

Changes in morphology and structure due to the cation substitution are hypothetically expected to have a direct impact on the biological performance (e.g., therapeutic ion leaching speed; cell adherence, viability, and proliferation) of SHA NPs. In this study, a series of preliminary in vitro biological assessments were carried out with the scope to shed further light on the effect of each cation.

The concentration of therapeutic ions to be released from HA into biological environments can be influenced by a number of independent or interconnected factors: composition, long-range order (e.g., crystalline quality, lattice alterations), ionic radii, specific surface area, and bond strength formed by ions in the host matrix lattice [93]. It is important to note the Ce, Mg, Sr, or Zn cation release tests were performed in the biomimetic complex organic–inorganic cell culture medium and not in distilled water or other inorganic simulated body media such as SBF or Tris-HCl (as is the case of most kindred studies) such as to increase the biological relevance of the yielded results.

The ICP-MS results (Figure 10) indicate that the crystalline quality played a prominent role on the dopant release. A material with higher crystalline quality possesses lower free energy and molecular mobility, which could result in more reduced degradation rates [94]. It is suggested that by increasing the long-range structural order, which could be achieved in the future by designed thermal-treatments (for instance), one could control the release of therapeutic (active) agents. On the other hand, the use of high levels of cationic substitution could benefit a more efficient therapeutic ion leaching into the intercellular fluids. Furthermore, a higher concentration of substituents could facilitate the HA decomposition into the more soluble β-TCP during thermal treatments and therefore foster a future advantageous route for the controlled fabrication of biphasic calcium phosphate bioceramics.

However, it needs to be stressed that significantly lower (with one order of magnitude) ion release concentrations were attained in the case of Sr- and Zn-substituted HAs (Figure 10a) with respect to Mg-HA (Figure 10b). It is suggested that besides the decrease of the crystalline quality of HA [95,96], other factors of influence could be at play and impact the therapeutic ion release rates in the biomimetic environments. For instance, the bond dissociation energy (enthalpy change, ΔHf) is higher for Ce–O (795 kJ·mol^−1^) and Sr–O (454 kJ·mol^−1^) with respect to Mg–O (394 kJ·mol^−1^) and Zn–O (284 kJ·mol^−1^) [75], and thus it is expected that once Ce–O and Sr–O bonds are formed within HA, they should be more stable and harder to break apart. The inferior release of Zn ions in comparison to Mg ones seems to be contradictory if one takes into account the lower dissociation energy of Zn–O bonds with respect to the Mg–O ones. Nevertheless, one should not dismiss the fact Zn does not substitute into the same Ca occupation sites as Mg (see Figure 8). The lower release of Zn ions with respect to Sr ions can be linked to the higher crystalline quality (Figure 7) and particle size (Figure 3) of Zn-HA as compared to Sr-HA.

For instance, for a better understanding of SHA interactions with bodily fluids and cells it would be advised to couple biofunctional assays with the determination of the temporal ion-release profiles. One technology that enables a real-time monitoring of cell behaviour with accuracy and good practice protocols without altering the cells (e.g., avoiding manipulating the medium or labelling) is RTCA, which could become a future asset for the study of the biological properties of such bioceramics. RTCA is a modern technique that has been applied with success in different areas of research (e.g., microbiology, pharmacology) [79,97,98,99,100] and has found its usefulness compared to other classical biological assays. To instigate bone formation, cells need to adhere and proliferate. In this respect, RTCA allows for noninvasive investigation and dynamic monitoring of the cell behaviour (e.g., cell adherence, proliferation, migration, and cytotoxicity) [79,97,98,99,100]. Briefly, adherent cells act as an insulator on the surface of the electrode and change the ionic medium of the electrode solution, impeding the flow of electric current, thereby increasing the impedance. This impedance value, plotted as a unitless parameter called the “cell index”, increases as cells continue to proliferate and then reaches a plateau as cells are approaching 100% confluence. The cell index value represents a quantitative measure of the growth status of the cells to be tested. The RTCA method has been found suitable to decrease the risk of contamination and to provide extra functionality compared with other classical methods (e.g., MTS, 3-(4,5-dimethylthiazol-2-yl)-2,5-diphenyltetrazolium bromide—MTT) [98]. As opposed to end-point analysis, it offers data at intermediary times, informative not only of the outcome of an effect (e.g., proliferation, death), but also of the trend line over time. However, surprisingly, RTCA analysis has been scarcely used to investigate the biological properties of HA [101] and was not yet reported for SHAs.

In the framework of this study, two types of cells were investigated via RTCA: osteoblast and endothelial cells. Osteoblasts play an important role in the bone matrix production, while endothelial cells are involved in the vascularisation process by regulating the release of vasodilator molecules [102]. None of the SHA materials elicited inferior cell adhesion values with respect to pure HA. The best composition-dependent adhesion (for both osteoblast and endothelial cells) was obtained in the case Mg-HAs (with emphasis on the HA-Mg0.5). This is in good agreement with other HA studies, which conveyed a Mg-mediated increased cell adhesion for osteoblast [103,104,105] and dermal fibroblast and keratinocyte [106] cells. However, to the best of our knowledge, there are no reports to date on the effect of Mg-HA on endothelial cells, and this positive effect (particularly at a low Mg dosage) needs to be emphasised. An adequate adhesion of the cells to the SHA bioceramics could have concomitantly an important impact on the other expressions of the cells (e.g., proliferation, differentiation) [107]. From proliferation point of view, the RTCA investigations indicate that all SHAs returned superior responses with respect to HA in the case of osteoblast cells, whilst this was valid only for Ce- and Sr-containing SHAs in the case of endothelial cells. Following RTCA assessments, one should consider whether a time-related, two-step intervention would mimic natural cell behaviour—firstly a Mg-based formulation to increase the local adherence of cells, followed by a long-term consolidation of cell proliferation based on Ce/Sr substitutes or a material with low degree of Mg and Zn substitution concomitant with a higher degree of Ce/Sr substitution would stimulate the proliferation of both osteoblasts and endothelial cells.

## 5. Conclusions

In summary, monophasic nanocrystalline pure and single-substituted HA materials can be synthesised using the coprecipitation method for concentrations of at least up to ~5 at.% for Mg, Sr, and Zn and of ~2.5 at.% in the case of Ce.

As expected, the crystalline lattice of HA was progressively weakened by the progressive incorporation of cation substituents (with the notable exception of Sr-HA). It was found that the therapeutic cation release speeds are conditioned by crystalline quality, bond dissociation energy, and/or occupation site of substituent (i.e., in the columnar or the screw axis Ca site) as interlinked influential factors. Mg was released at the highest concentrations with respect to the other cations as three important criteria were met: moderate crystalline quality of Mg-HAs, low dissociation energy of Mg–O, and a preference of Mg to occupy the Ca(I) (columnar) lattice sites.

Most substituted HA nanopowders presented good cytocompatibility, comparable or even superior with respect to the pure HA and the biological control. Moreover, RTCA was applied for the first time for the study of substituted HAs and has allowed for the discrimination of the behaviour of two cell culture types (osteoblast and endothelial), representative for two of the most important processes in the bone healing process: colonization with osteoblasts with callus neoformation and vascularization; responses were promoted by various types and contents of cation substituents. The results suggest that Zn and Mg ions are useful as a first intervention for cell adherence, whereas Ce and Sr could sustain a long-term cell proliferation.

In the near future, special focus will be placed on the design and integration of multisubstituted HA-based bioceramics with controlled therapeutic ion release into implant coatings and bone graft substitutes (porous scaffolds) and on their insightful mechanical and complex biological evaluation.

## Data Availability

All data generated or analysed during this study are included in the published article. The raw data can be made available from the authors upon reasonable request.
